# Triage of referrals in a child and adolescent mental health service in Qatar: reducing waiting times and promoting needs-based prioritisation

**DOI:** 10.1192/bji.2021.10

**Published:** 2021-08

**Authors:** Yasser Saeed Khan, Mahmoud Al-Shamlawi, Lazarus Phiri, Majid Alabdulla

**Affiliations:** 1 Consultant Child and Adolescent Psychiatrist and Medical Lead, Child and Adolescent Mental Health Service, Hamad Medical Corporation, Doha, Qatar. Email ykhan5@hamad.qa; 2 Charge Nurse, Child and Adolescent Mental Health Service, Hamad Medical Corporation, Doha, Qatar; 3 Clinical Nurse Specialist, Child and Adolescent Mental Health Service, Hamad Medical Corporation, Doha, Qatar; 4 Senior Consultant Psychiatrist and Chair of Mental Health Services, Hamad Medical Corporation, Doha, Qatar; 5 College of Medicine, Qatar University, Doha, Qatar

**Keywords:** Triage, child and adolescent mental health service, referrals, out-patient appointments, waiting list

## Abstract

This paper summarises the impact of a new triage process on referral prioritisation and waiting times in a community specialist child and adolescent mental health service (CAMHS) in Qatar. The process involves initial review of referrals by a CAMHS nurse to ensure that there is adequate clinical information, obtaining additional information from patients/families and referring clinicians by the psychiatric triage team, when necessary, followed by prioritisation and allocation of accepted referrals. The new process reduced the acceptance of inappropriate referrals, ensured prioritisation of referrals and significantly improved the service's compliance with waiting-time deadlines.

A seminal moment in the transformation of Qatar's healthcare system was the launch of the National Health Strategy 2018–2022 under the theme ‘Our Health, Our Future’ in 2018. One purpose of this drive was to further improve the capacity and performance of the healthcare system through efficient use of available resources. The initiative described in this paper was taken, among many others, to support Qatar's National Health Strategy.

## Background

Most healthcare services face the challenging task of reducing waiting times to comply with their specified time frames. A long waiting time is associated with a higher rate of non-attendance in out-patient clinics.^1^ It is not uncommon for out-patient child and adolescent mental health services (CAMHS) to have long waiting lists, which can become a source of significant distress for children, young people and their families. A number of initiatives have been described to reduce waiting times and improve attendance at CAMHS out-patient clinics.^2–4^

Triage of referrals is the most common entry point into a healthcare service and is considered a crucial component in the care pathway. When adopted effectively, the process of triage cuts waiting times significantly and ensures that the needs of patients and their families are met safely and appropriately. A UK study showed that triage for CAMHS is feasible and that it resulted in reduced waiting times for a first appointment.^5^ Referral quality is also crucial to efficient patient flow and it should therefore be necessary, appropriate, timely and well communicated.^6^

The CAMHS of Hamad Medical Corporation in Qatar is a community-based out-patient service providing help and support to children and young people with mental and behavioural disorders and their families. The CAMHS team has adopted the multidisciplinary way of working to ensure that the needs of patients and families are addressed holistically. It comprises psychiatrists, psychologists, psychiatric nurses, occupational therapists, speech and language therapists, dietitians and social workers. The service received 912 new referrals during 2018 and it was anticipated that, owing to rising demand, this number would increase further in the following years. The challenge of meeting the waiting times, both for routine and urgent appointments, was consequently growing considerably.

## Historical practice and creation of triage team

The need to develop an effective triage team within the CAMHS was identified in early 2019 to ensure the suitability of accepted referrals, needs-based prioritisation and compliance with waiting times. A broad consensus was reached regarding the need to shift from the historical practice of using clinical information enclosed in the referral only, irrespective of its comprehensiveness, to make a clinical judgement regarding its appropriateness, level of urgency and allocation to the most suitable clinician within the multidisciplinary team. Instead, it was agreed to obtain additional information from the referring clinician and patients/carers, where appropriate, before processing various aspects of a referral.

The practice of allocation of the majority of referrals to psychiatrists for initial assessments was also abandoned. It was replaced by allocating referrals strictly on the basis of individual needs of children/young people and their families to ensure a high standard of care and also to utilise the skill range and resources across the multidisciplinary team effectively. The need to involve psychiatric nurses in the process of triage of referrals was also recognised.

## Process and implementation

The process started with the development of a screening instrument to enable staff to obtain additional information in a structured and consistent manner. This instrument was based on existing recognised tools, mainly the UK Mental Health Triage Scale and the Canadian Triage and Acuity Scale, and concepts adopted globally. This was followed by providing further training and teaching to staff to enhance their skills to obtain crucial additional clinical information in case of inadequate referrals and to complete a thorough risk assessment using the screening form. This was achieved through senior clinicians in the team providing face-to-face teaching and training.

The CAMHS senior leadership agreed that specialist psychiatric nurses, middle-grade doctors (specialists and fellows) and consultant psychiatrists would be part of the triage team, with the expectation that they would meet three times weekly, operating on a monthly roster.

Once a referral is received, it is reviewed by the CAMHS nurse, who then has a discussion with the middle-grade doctor and a consultant psychiatrist.

If the referral has no adequate clinical information to establish suitability for the service, prompt communication is made with the referring clinician asking them to provide the required information. If the referral is deemed suitable but the information is still insufficient to prioritise and allocate it appropriately, telephone contact is made with the young person/parents to obtain additional information. This process of contacting young people and families, referred to as screening, mainly serves the purpose of establishing the urgency of referral further and identifying the most suitable clinician from the multidisciplinary team for allocation. The information gathered during the screening process includes relevant aspects of clinical history and risk issues and is obtained using the standard screening form to ensure consistency.

Referrals are therefore deemed emergency, urgent or routine based on information obtained from the referrer and patient/family. Emergency referrals are either seen within 24 h in CAMHS, where possible, or asked to report to the emergency department of Hamad General Hospital or the paediatric emergency centre to be assessed by the psychiatric consultation and liaison team or the on-call psychiatric team. All referrals deemed urgent are offered appointments for initial assessments in the CAMHS out-patient clinic within 7 calendar days and routine referrals within 42 calendar days of their receipt.

## Impact on service standards

A service review was undertaken in October 2019 to evaluate the impact of the triage process on service effectiveness. We were interested to know whether the new process had resulted in better prioritisation of referrals and any change to waiting times. The review also included a clinical audit in which current performance was measured against the service standard of compliance with the time frames for both routine (within 42 calendar days) and urgent (within 7 calendar days) new-patient out-patient appointments.

**Fig. 1:**
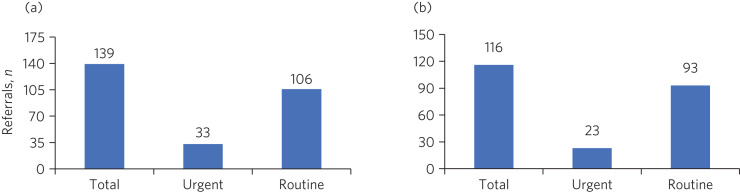
(a) Prioritisation of referrals in January–March 2018. (b) Prioritisation of referrals in July–September 2019.

We compared two 3-month periods. Data for the first 3 months of 2018 (pre-triage period) were compared with data for the 3 months immediately prior to when the audit was conducted (post-triage period) to enable us to assess the actual impact, if any, of the novel process. A total of 168 new referrals were received during July–September 2019, compared with 194 during January–March 2018. The percentage of referrals deemed appropriate and therefore accepted by the service reduced by 3%, i.e. from 139 out of 194 (72%) referrals during January–March 2018 (Fig. 1(a)) to 116 out of 168 (69%) during July–September 2019 (Fig. 1(b)). The triage team screened 73 out of 116 (63%) accepted referrals. Referrals that were not screened by the triage team had sufficient clinical information to be allocated for urgent or routine appointments.

**Fig. 2:**
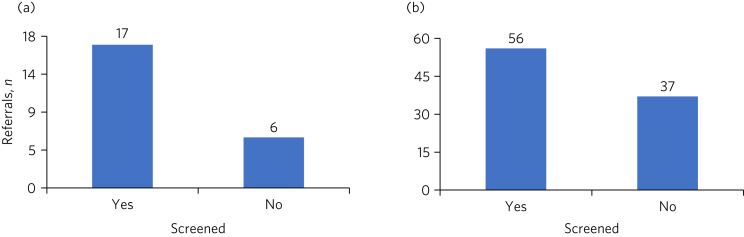
July–September 2019: (a) proportion of urgent referrals that had received prior screening; (b) proportion of routine referrals that had received prior screening.

The percentage of referrals allocated for urgent appointments (i.e. within 7 calendar days of receipt of a referral) reduced from 23.7% in 2018 to 19.8% in 2019 following the introduction of the new triage process. Almost three-quarters of the referrals allocated for urgent appointments had received prior screening (Fig. 2(a)) to establish their urgency and to ensure effective prioritisation. The triage was able to ascertain, through obtaining additional information from the young person/family (screening) as well as the referring clinicians, that some of these referrals had no justifiable clinical rationale to be deemed urgent and were therefore changed to routine referrals.

A slight increase (from 76.2 to 80.1%) was noted in the proportion of referrals allocated for routine appointments (to be seen within 42 calendar days). The triage team had screened 60% of these referrals before they were allocated for routine appointments (Fig. 2(b)). A number of routine referrals were deemed urgent by the triage team after screening and were instead prioritised for urgent appointments.

**Fig. 3:**
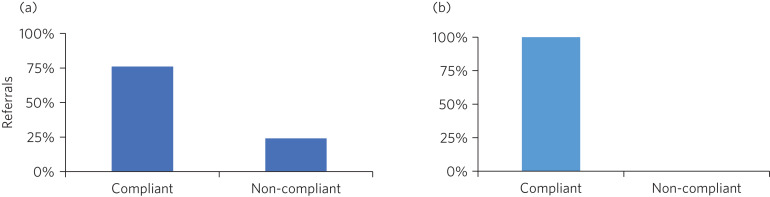
(a) The service's compliance with waiting time deadlines for urgent referrals in January–March 2018. (b) Compliance with waiting time deadlines for urgent referrals in July–September 2019.

The most significant statistic was the service's rate of compliance with the locally agreed time frames (service standards) for urgent and routine appointments. The rate of compliance for all accepted referrals (urgent and routine combined) improved from 85.6 to 90.5%. More importantly, the rate of compliance for urgent referrals saw a remarkable increase, from 75.7% in 2018 (Fig. 3(a)) to 100% in 2019 (Fig. 3(b)) as a result of effective screening and prioritisation of referrals. All 23 patients whose referrals were deemed urgent during the period July–September 2019 were offered initial appointments within 7 calendar days, as per the service standard. The compliance for routine referrals almost remained the same, i.e. 88.6% in 2018 and 88.1% in 2019.

## Conclusions

• Effective triage of referrals has the potential to improve compliance with time deadlines (service standards) and therefore to reduce the waiting-list duration.
• Triage of referrals can reduce the acceptance of inappropriate referrals and promote effective prioritisation and risk management through an efficient screening process.
• Allocation of referrals through appropriate triage to the most suitable clinician within a multidisciplinary team improves the compliance of a service with its set time deadlines and enhances the quality of care provided to children, young people and their families by providing needs-based interventions at the outset.
• Psychiatric nurses can play a crucial role in the effective triage of referrals within specialist mental health services when given the required support from their medical colleagues.

## Data Availability

Data available upon request from the corresponding author.

## Supplementary material

For supplementary material accompanying this paper visit https://doi.org/10.1192/bji.2021.10. click here to view supplementary material
